# Fucoidan inhibits apoptosis and improves cardiac remodeling by inhibiting p53 transcriptional activation through USP22/Sirt 1

**DOI:** 10.3389/fphar.2023.1164333

**Published:** 2023-05-30

**Authors:** Shuai Wang, Jie Bai, Yilin Che, Weikun Qu, Jing Li

**Affiliations:** ^1^ Second Afliated Hospital of Dalian Medical University, Dalian, China; ^2^ School of Public Health, Dalian Medical University, Dalian, China; ^3^ The 1st Department of Thoracic Medical Oncology, Second Affiliated Hospital of Dalian Medical University, Dalian, China; ^4^ Department of OPO Office, Second Affiliated Hospital of Dalian Medical University, Dalian, China; ^5^ Department of Cardiology, Institute of Heart and Vascular Diseases, Second Affiliated Hospital of Dalian Medical University, Dalian, China

**Keywords:** USP22, fucoidan, cardiac remodeling, Sirt 1, apoptosis

## Abstract

**Background:** Humans with hypertensive heart disease are more likely to experience heart failure, arrhythmia, myocardial infarction, and sudden death, and it is crucial to treat this condition. Fucoidan (FO) is a natural substance derived from marine algae that has antioxidant and immunomodulatory activities. FO has also been shown to regulate apoptosis. However, whether FO can protect against cardiac hypertrophy is unknown.

**Methods:** We investigated the effect of FO in hypertrophic models *in vivo* and *in vitro*. C57BL/6 mice were given an oral gavage of FO (300 mg/kg/day) or PBS (internal control) the day before surgery, followed by a 14-day infusion of Ang II or saline. AC-16 cells were treated with si-USP22 for 4 h and then treated with Ang II (100 nM) for 24 h. Systolic blood pressure (SBP) was recorded, echocardiography was used to assess cardiac function, and pathological changes in heart tissues were assessed by histological staining. Apoptosis levels were detected by TUNEL assays. The mRNA level of genes was assessed by qPCR. Protein expression was detected by immunoblotting.

**Results:** Our data showed that USP22 expression was lowered in Ang II-infused animals and cells, which could promote cardiac dysfunction and remodeling. However, treatment with FO significantly upregulated the expression of USP22 and reduced the incidence of cardiac hypertrophy, fibrosis, inflammation, and oxidative responses. Additionally, FO treatment lowered p53 expression and apoptosis while increasing Sirt 1 and Bcl-2 expression.

**Conclusion:** By reducing the level of Ang II-induced apoptosis through the regulation of USP22/Sirt 1 expression, FO treatment might improve cardiac function. According to this study, FO might be potential targeted approach for treating heart failure.

## Introduction

Diastolic dysfunction (CHF-D) and left ventricular hypertrophy (LVH) are crucial indicators of hypertensive heart disease (Slivnick and Lampert, 2019). Because individuals with hypertensive heart disease are more likely to experience heart failure, arrhythmia, myocardial infarction, and sudden death, it is crucial to cure this condition. Anti-hypertensive therapy aims to lower blood pressure (BP) and stop the pathophysiological processes that cause LVH and CHF-D that are not dependent on blood pressure (Diamond and Phillips, 2005; Shimizu I and Minamino, 2016). The renin-angiotensin system (RAS) controls salt intake, vasoconstriction, potassium excretion, blood pressure, and other physiological processes (Lev-Ran and Porta, 2005). The main RAS effector molecule is angiotensin II (Ang II). This factor raises blood pressure, influences the renal tubules to retain sodium and water, and increases the release of aldosterone from the adrenal glands. Ang II is a powerful vasoconstrictor that also has proliferative, inflammatory, and fibrotic effects (Benigni et al., 2010). Angiotensinogen type 1 receptors (AT1Rs), which are widely distributed in all organs, including the heart and vascular system, mediate the majority of the known physiological activities of Ang II. Thus, inhibiting AT1R-mediated activation of signaling is critical for blocking hypertensive heart disease.

By attaching or removing ubiquitins to substrate proteins, ubiquitination and deubiquitination are critical posttranslational modifications of metabolic enzymes that control their breakdown, delocalization, and activation in cells (Eletr ZM and Wilkinson KD, 2014). Cardiovascular diseases are more likely to develop when ubiquitination and deubiquitination are dysregulated, which is directly connected to lipid metabolism in cells (Gu et al., 2022). Deubiquitinating enzymes (DUBs) have a critical role in cardiovascular illnesses such as cardiac hypertrophy, myocardial infarction, atrial fibrillation, and heart failure, according to recent research (Komander D., 2010; Komander D and Rape M., 2012; Kliza and Husnjak, 2020). USP22, a member of the ubiquitin-specific protease (USP) subfamily of DUBs, has been linked to both human and mouse malignancies and placental development in mice (Melo-Cardenas J et al., 2016). According to a recent study, USP22 may prevent cardiac ischemia‒reperfusion damage by preventing cardiomyocyte death (Ma S et al., 2020). The nicotinamide adenine dinucleotide (NAD)-dependent protein deacetylase sirtuin-1 (Sirt 1) is specifically deubiquitinated by USP22, and this deubiquitination leads to the stabilization of the Sirt1-repressed tumor protein p53, affecting transcriptional and proapoptotic activities (Lin Z et al., 2012). Sirt1 was shown to contribute to myocardial ischemia and reperfusion damage, as well as vascular endothelial dysfunction, safeguarding mitochondrial function, inhibiting oxidative stress, and relieving the inflammatory response (Luo G et al., 2019). Therefore, the goal of this study was to determine whether USP22 and Sirt 1 were involved in the molecular processes underlying the control of apoptosis in Ang II-induced cardiac remodeling.

Fucoidan (FO) is a fucose-enriched sulfated polysaccharide that is mostly produced by brown algae and has been extensively used as a dietary supplement and health food because of its many positive effects, including anti-inflammatory, anticancer, and antidiabetic effects (Li and Ye, 2008; Zhang SM et al., 2015; Fitton HJ et al., 2019). An FO preparation called ‘Haikun Shenxi capsules’ was approved in China in 2003, and its clinical use as a treatment for chronic renal failure has been described (Fitton HJ et al., 2019). This preparation has been shown to alleviate diabetes-induced kidney fibrosis by increasing the levels of USP22 and inducing deubiquitination of the Sirt1 protein through overexpression (Yu et al., 2020). However, the molecular processes underlying the functions of USP22 and Sirt1 in Ang II-induced ventricular hypertrophy are unknown.

In this study, we used FO as a protective agent to investigate its effects on USP22/Sirt 1 in Ang II-induced cardiac hypertrophy and aimed to highlight a novel targeted approach to treat heart failure.

## Materials and methods

### Antibodies and chemicals

Fucoidan (FO) was obtained from Med Chem Express (HY-132179), the average molecular weight is 220–300 kDa, polysaccharide, content of this fucan is approx (20%–23%), sulphate approx (24%–30%). Anti-USP22 (1:800, ab195289) and calcineurin A (CaNA, 1:800, ab71149) primary antibodies were from Abcam; anti-Sirt 1 (1:800, WL02995) and anti-transforming growth factor-beta 1 (TGF-β1) (1:800, WL02998) were from Wanleibio; anti-CD68 (1:600, bs-1432R) and anti-NADPH-Oxidase 4 (NOX4) (1:600, bs-1091R) were from Bioss Antibodies; anti-p53 (1:1000, #2524) and anti-Bcl-2 (1:500, #3498) were from Cell Signaling Technology; and anti-GAPDH (1:2000, AP0063) and anti-β-tubulin (1:2000, AP0064) were from BIOWORLD. Wheat germ agglutinin (WGA) was purchased from Vector Laboratories. Dihydroethidium (DHE) was purchased from BIOFOUNT. The TUNEL Apoptosis Detection Kit (Cat# 40308) was purchased from YEASEN.

### Animal study

Four groups of forty wild-type (WT) C57BL/6 male mice (8 weeks old) were used. Using osmotic mini-pumps (Alzet Model 1007D or 1002, DURECT), the mice were infused for 7 or 14 days with normal saline or Ang II (1,000 ng/kg/min, Aladdin) (Bai et al., 2022). The mice were given an oral gavage of FO (300 mg/kg/day) or PBS (internal control) the day before surgery, followed by a 14-day infusion of Ang II or saline (Yu et al., 2020). All mice received an intraperitoneal injection of 2.5% tribromoethanol (0.02 mL/g, Sigma‒Aldrich) to induce anesthesia after receiving therapy for 14 days. The hearts were removed and used for future research.

The animal experimental procedures were approved by the Animal Experimental Ethics Committee of Dalian Medical University, and extensive efforts were made to minimize the distress of the included animals.

## Monitoring of blood pressure and cardiac function

The blood pressure of mice in each group was measured by a noninvasive blood pressure automatic measurement system (BP-300A, Chengdu Taimeng Software Co., Ltd.). Mice in each group were weighed and anesthetized with 1.5% isoflurane. Cardiac function was monitored using a 70 MHz probe (Vevo 3,100 System, FUJIFILM).

### Histopathology and immunohistochemical analysis

Myocardial tissue was fixed with 4% paraformaldehyde, embedded in paraffin or optimal cutting temperature compound (OCT) and sectioned (5 μm). Cardiac tissue sections were immersed in hematoxylin for 3 min, stained with eosin for 5 min, and then sealed with neutral gel. Masson staining was used to observe myocardial fibrosis. Cardiomyocyte (CM) hypertrophy was detected by WGA staining, and reactive oxygen species (ROS) levels were detected by DHE staining. Four fields were randomly selected in the stained sections and observed microscopically.

For immunohistochemical staining, heart tissue sections were incubated with an anti-CD68 antibodies (1:200), and then development was performed with DAB as previously described (Bai et al., 2022).

### Cell culture and treatment

AC-16 cells were cultured in DMEM/F12 with 10% FBS and 1% penicillin/streptomycin. The cells were starved for 2 h, treated with si-USP22 and si-control for 4 h, and then treated with Ang II (100 nM) or saline for 24 h. Similarly, cells were starved for 2 h, treated with FO (60 μg/mL) for 4 h, and then treated with Ang II (100 nM) or saline for 24 h (Zhang et al.,. 2015).

### Heart tissue and cell RNA extraction and RT‒qPCR detection

Using TRIzol reagent, we isolated total RNA from cardiac tissue and cells (Invitrogen). Using superscript II, the first strand of cDNA was produced from total RNA (2 μg) (Invitrogen). Real-time quantitative PCR was used to identify the mRNA levels of USP22, Atrial natriuretic factor (ANF), brain natriuretic peptide (BNP), collagen I, collagen III, Interleukin-1β (IL-1β), Interleukin-6 (IL-6), NADPH-Oxidase 2 (NOX2) and NOX4. These data were normalized to GAPDH. Sango Biotech provided the primers. Table 1 shows the primer details.

**TABLE 1:** The details of primers used in RT-qPCR.

Gene	Forward primer (5′-3′)	Reverse primer (5′-3′)
USP22	CAT​GAC​CCC​TTT​CAT​GGC​CT	GAT​GTT​CTG​GTG​ACG​GGT​GT
ANF	CAC​AGA​TCT​GAT​GGA​TTT​CAA​GA	CCT​CAT​CTT​CTA​CCG​GCA​TC
BNP	GAA​GGT​GCT​GTC​CCA​GAT​GA	CCA​GCA​GCT​GCA​TCT​TGA​AT
Collagen I	GAG​TAC​TGG​ATC​GAC​CCT​AAC​CA	GAC​GGC​TGA​GTA​GGG​AAC​ACA
Collagen III	TCC​CCT​GGA​ATC​TGT​GAA​TC	TGA​GTC​GAA​TTG​GGG​AGA​AT
NOX2	ACC​GGG​TTT​ATG​ATA​TTC​CAC​CT	GAT​TTC​GAC​AGA​CTG​GCA​AGA
NOX4	CAG​ATG​TTG​GGG​CTA​GGA​TTG	GAG​TGT​TCG​GCA​CAT​GGG​TA
IL-1β	TGC​CAC​CTT​TTG​ACA​GTG​ATG	TGA​TGT​GCT​GCT​GCG​AGA​TT
IL-6	TGA​TGG​ATG​CTA​CCA​AAC​TGG​A	TGT​GAC​TCC​AGC​TTA​TCT​CTT​GG
GAPDH	GGT​TGT​CTC​CTG​CGA​CTT​CA	GGT​GGT​CCA​GGG​TTT​CTT​ACT​C

### Western blotting

A total protein extraction kit (BC3711, Solarbio) was used to extract the total protein from cardiac tissue and cells, and a BCA kit from Thermo Scientific was used to quantify the protein concentrations. Equal amounts of protein (25 μg) were placed on a PVDF membrane after being separated by 10% or 12% SDS‒PAGE. The membrane was incubated with the binding antibody (Thermo Scientific) overnight at 4 °C. With the aid of the ECL Light Chemiluminescence Kit, the bands were discovered (Epizyme Biomedical Technology). We used β-tubulin and GAPDH as internal controls.

#### TUNEL assay

The TUNEL apoptosis detection kit was used to detect apoptosis in frozen cardiac tissue sections and cells after they had been dried at room temperature for 30 min, fixed with 4% paraformaldehyde, and washed with PBS solution. In the stained sections, four fields of view were chosen at random for microscopic examination.

### Statistical analysis

Statistical analysis of the data was performed with GraphPad Prism 9.0. First, a normalcy test was conducted. Student’s t-test or one-way ANOVA was used as necessary if all groups satisfied the requirements for normality and the intergroup variances were equal. If the aforementioned requirements were not met, the nonparametric Mann‒Whitney *U* test was used. The threshold for significant differences was *p* < 0.05. The results are shown as the mean ± SD.

## Results

### USP22 expression was downregulated by Ang II

**FIGURE 1:**
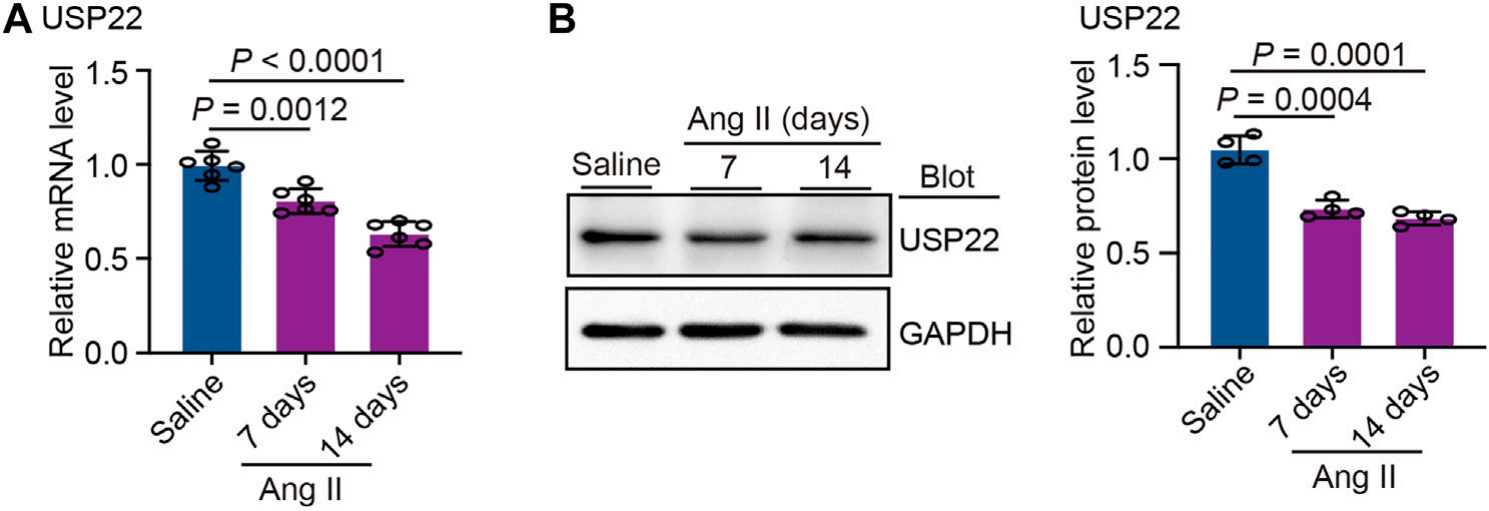
Ang II reduced the expression of USP22. The WT C57BL/6 mice were infused with Ang II (1,000 ng/kg/min) or saline for 7 or 14 days. **(A)** The mRNA expression of USP22 in heart at day 7 and 14 of Ang II infusion (*n* = 6 per group); **(B)** Western blot analysis of USP22 protein in hearts (left) and quantification of protein bands (right, *n* = 4).

We initially measured the levels of USP22 in the heart 7 and 14 days after Ang II infusion to better understand the role of USP22 in Ang II-induced cardiac remodeling. The levels of USP22 mRNA dramatically decreased after 7 and 14 days of Ang II infusion, as shown in Figure 1A. Western blot analysis revealed that following Ang II infusion, USP22 expression was downregulated (Figure 1B). Therefore, the incidence of Ang II-induced cardiac remodeling may be significantly influenced by the reduced expression of USP22.

### USP22 improves Ang II-Induced Hypertension and cardiac insufficiency

**FIGURE 2:**
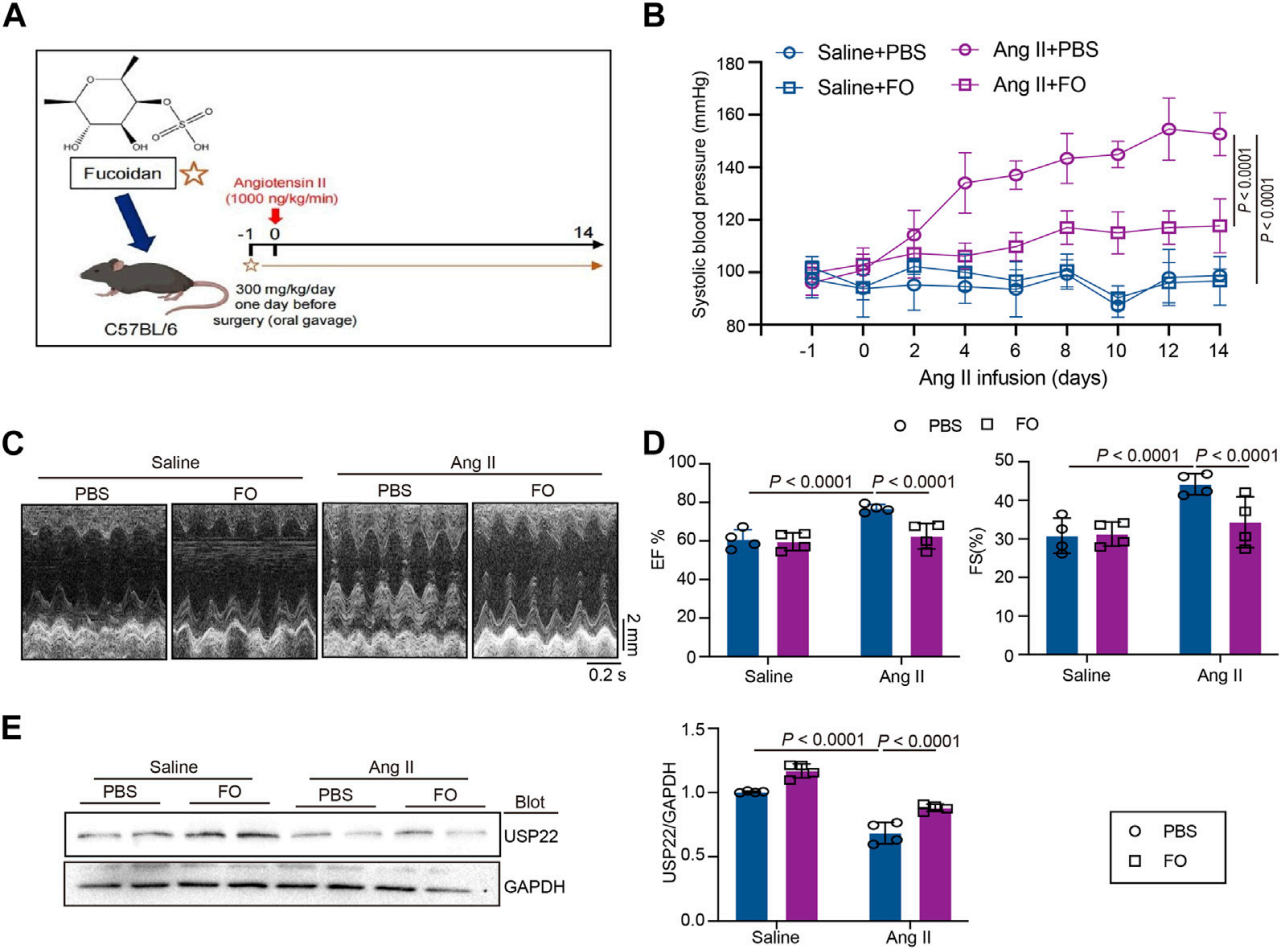
FO treatment increased cardiac function and decreased SBP by upregulating the expression of USP22. The WT C57BL/6 mice were treated with Fucoidan (FO) (300 mg/kg/day) 1 day before surgery, and then the mice were infused with Ang II (1,000 ng/kg/min) or saline for 14 days. **(A)** The structure of FO and a protocol of administration of FO in Ang II-infused model of cardiac remodeling. **(B)** The systolic blood pressure (SBP) was measured 1 day before surgery signed as −1 day and then every 2 days after Ang II infusion (*n* = 6); **(C)** M-mode echocardiography of LV chamber at 14 days (*n* = 4); **(D)** Measurement of EF% and FS% (*n* = 6); **(E)** Western blot analysis of USP22 protein in hearts (left), and quantification of protein bands (right, *n* = 4).

The mice were given an oral gavage of FO (300 mg/kg/day) the day before surgery and then received an Ang II infusion for 14 days (Figure 2A). Systolic blood pressure (SBP) was measured in each group, as shown in Figure 2B, and the results revealed that Ang II infusion significantly increased SBP, while FO treatment significantly lowered Ang II-induced high SBP in comparison to the Ang II + PBS group. The echocardiography results demonstrated that FO greatly reduced Ang II-induced cardiac dysfunction by raising the levels of ejection fraction (EF%) and fractional shortening (FS%) (Figures 2C, D). We next examined USP22 expression, and the results showed that after receiving FO, animals given Ang II had increased USP22 protein expression (Figure 2E). These findings suggested that FO therapy could increase USP22 expression, which might reduce SBP and cardiac dysfunction.

### USP22 inhibits cardiac hypertrophic in Ang II-infused hearts

**FIGURE 3:**
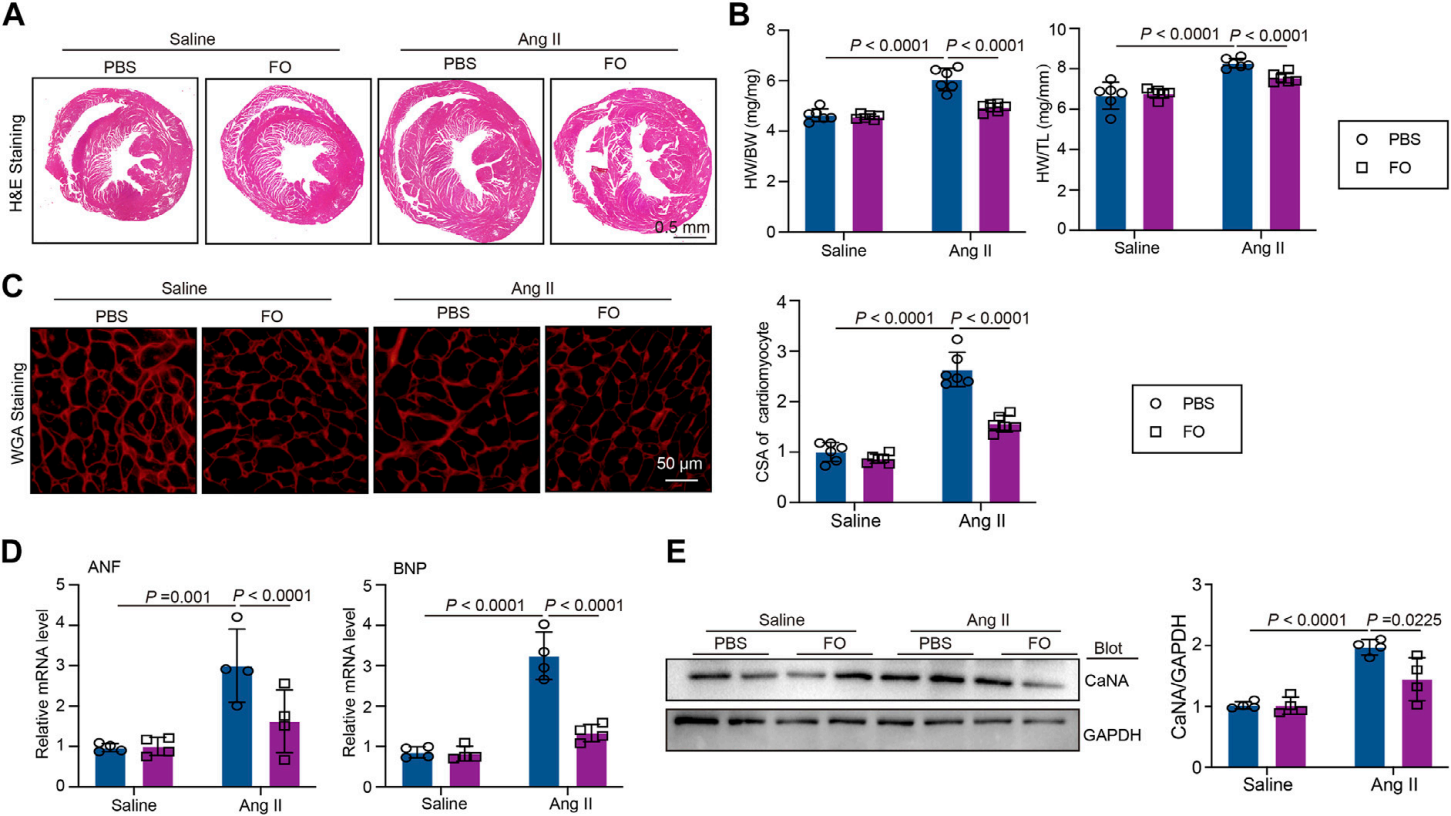
FO treatment improves Ang II-induced cardiac hypertrophy. The WT C57BL/6 mice were treated with Fucoidan (FO) (300 mg/kg/day) 1 day before surgery, and then the mice were infused with Ang II (1,000 ng/kg/min) or saline for 14 days. **(A)** H & E staining of heart sections in each group (*n* = 4); **(B)** The ratio of heart weight to body weight (HW/BW) and heart weight to tibial length (HW/TL, *n* = 6); **(C)** WGA staining of heart sections in each group (left, *n* = 6), the quantitation of CSA of cardiomyocyte (right, *n* = 6); **(D)** The mRNA level of ANF and BNP in heart sections of each group (*n* = 4); **(E)** Western blot analysis of CaNA protein expression in hearts (left), and quantification of protein bands (right, *n* = 4).

The role of USP22 in Ang II-induced cardiac hypertrophy and the oxidative response was then investigated. Left ventricular thickness, heart weight/body weight (HW/BW), and heart weight/tibia length (HW/TL) were significantly increased 2 weeks after Ang II infusion, and FO therapy could reduce these effects (Figures 3A, B). Additionally, we used WGA staining to show how the cross-sectional areas of the myocytes changed in each group. The findings demonstrated that FO therapy reduced myocyte size in comparison to that in the Ang II + PBS group (Figure 3C). In Ang II-infused animals, the expression of hypertrophic markers ANF and BNP was reduced after FO therapy (Figure 3D). Similarly, in Ang II-infused mice, FO reduced the production of CaNA protein, which are involved in the hypertrophic signaling cascade (Figure 3E).

### Addition of USP22 reduces Ang II-induced cardiac inflammation and fibrosis

**FIGURE 4:**
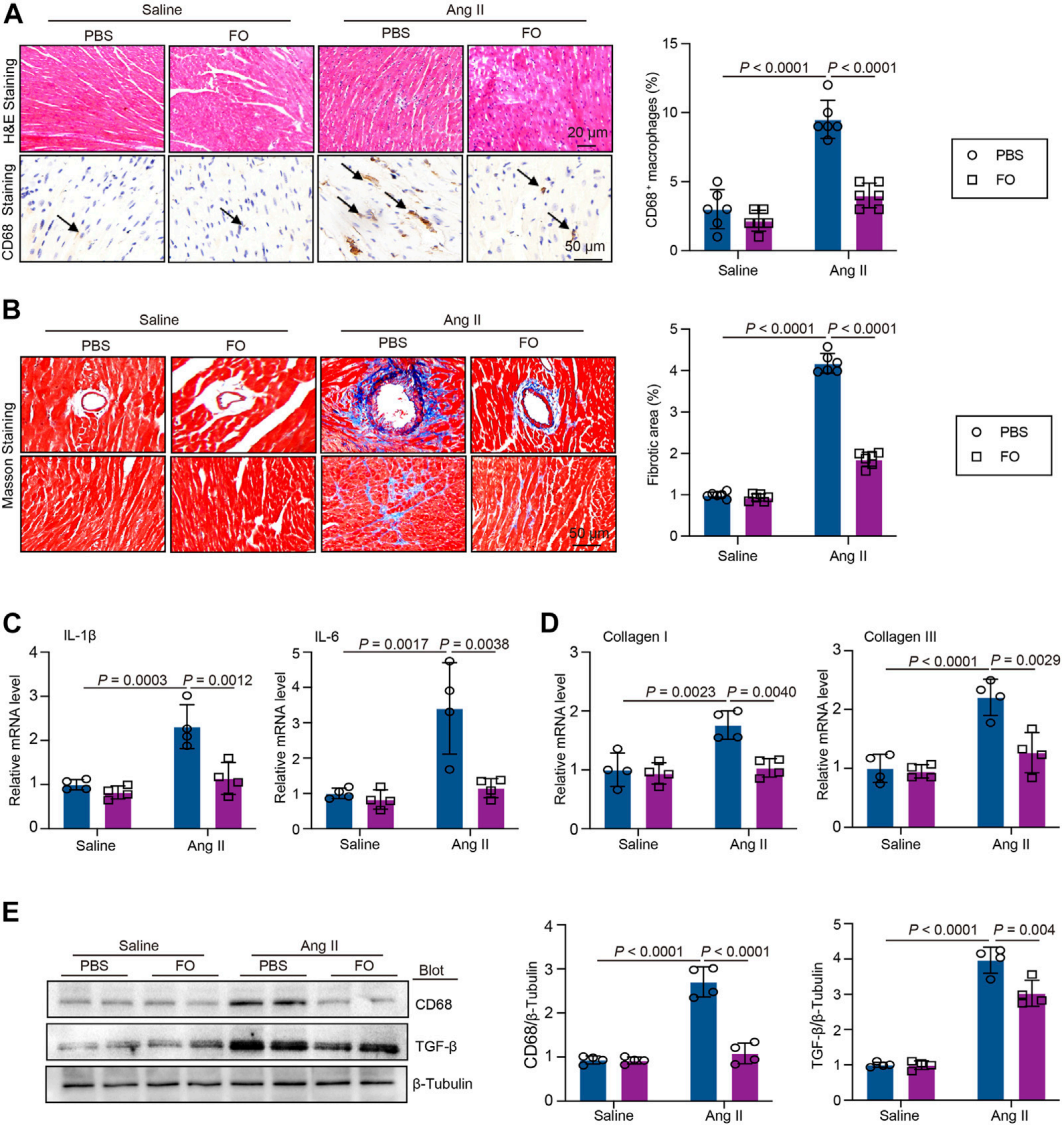
FO treatment decreased Ang II-induced cardiac inflammatory cell and collagen deposition. The WT C57BL/6 mice were treated with Fucoidan (FO) (300 mg/kg/day) 1 day before surgery, and then the mice were infused with Ang II (1,000 ng/kg/min) or saline for 14 days. **(A)** H & E and CD68 immunohistochemical staining of heart sections in each group (left, *n* = 4), the quantification CD68 positive macrophages (right, *n* = 6); **(B)** Masson staining of heart section s in each group (*n* = 6), the quantification of fibrotic area in each heart section (*n* = 6); **(C)** The mRNA level of IL-1β and IL-6 in heart sections of each group (*n* = 4); **(D)** The mRNA level of collagen I and collagen III in heart sections of each group (*n* = 4); **(E)** Western blot analysis of CD68 and TGF-β protein expression in hearts (left), and quantification of protein bands (right, *n* = 4).

We administered FO to mice to increase USP22 expression to confirm the involvement of USP22 in Ang II-induced inflammatory damage and cardiac fibrosis. The results showed that after FO treatment, inflammatory CD68^+^ cells in Ang II-infused hearts significantly decreased (Figure 4A). In the majority of cardiac disorders, myocardial fibrosis is a histological marker of structural change. An increase in USP22 in Ang II-infused mice reduced collagen deposition, as shown by Masson staining (Figure 4B). The mRNA levels of IL-1β, IL-6, collagen I, and collagen III were next examined, and we discovered that FO dramatically reduced the expression of inflammatory and fibrosis markers in mice that had received Ang II (Figures 4C, D). Additionally, Ang II increased the expression of CD68 and TGF-β1 relative to saline infusion, which was also inhibited in mice treated with FO (Figure 4E).

USP22 Protects Against Ang II-induced Oxidative Reactions, and Apoptosis Through the Sirt 1/p53 Axis.

**FIGURE 5:**
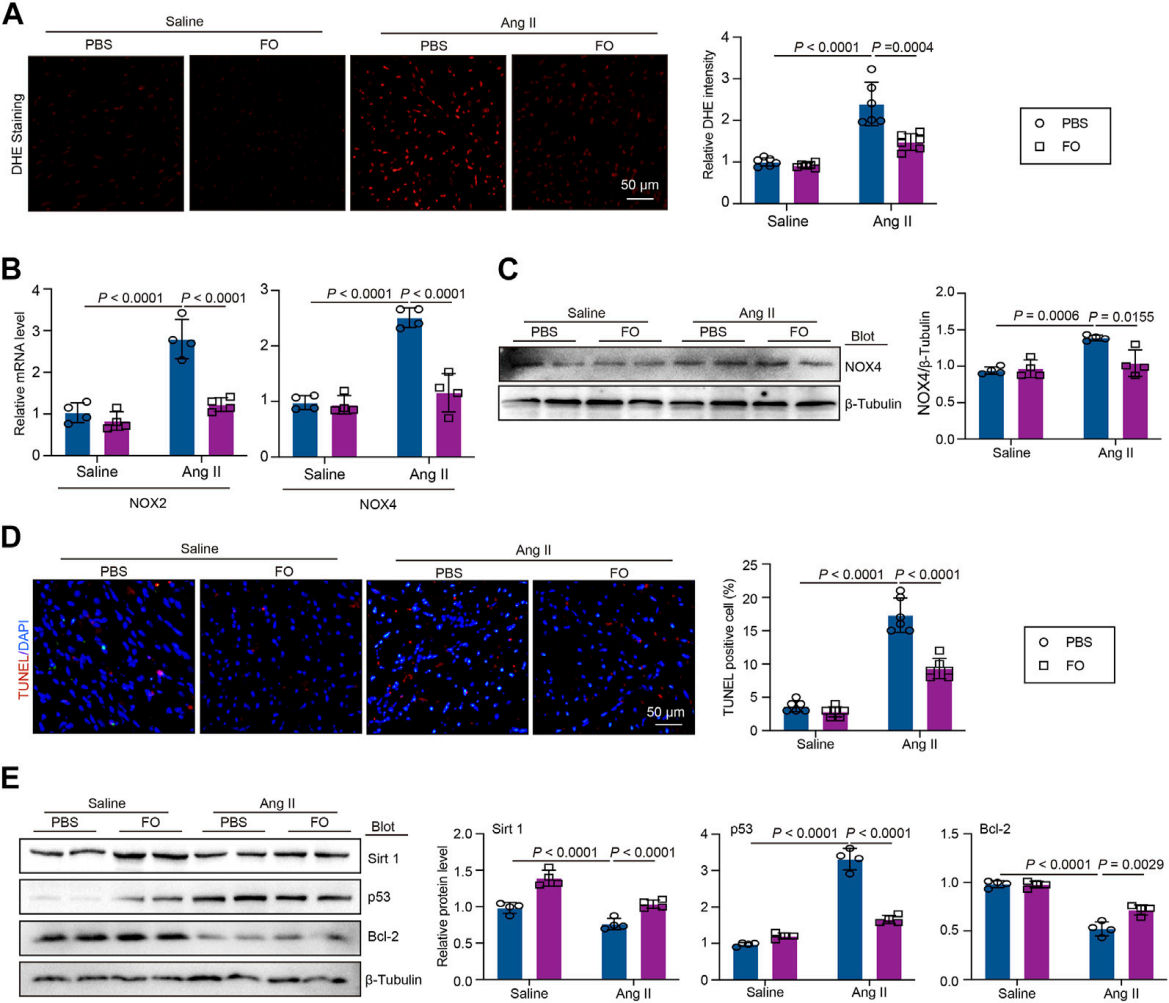
FO treatment reduced Ang II-induced cardiac oxidative stress and apoptosis by regulating the expression Sirt 1 and p53. The WT C57BL/6 mice were treated with Fucoidan (FO) (300 mg/kg/day) 1 day before surgery, and then the mice were infused with Ang II (1,000 ng/kg/min) or saline for 14 days. **(A)** DHE staining of cardiac sections in each group (left, *n* = 6), the quantification of DHE intensity (right, *n* = 6); **(B)** The mRNA level of NOX2 and NOX4 in heart sections of each group (*n* = 4); **(C)** Western blot analysis of NOX4 protein expression (left), and quantification of protein bands (right, *n* = 4). **(D)** TUNEL staining of cardiac sections in each group (left, *n* = 4), the quantification of TUNEL positive cell (right, *n* = 6); **(E)** Western blot analysis of Sirt 1, p53 and Bcl-2 protein expression in hearts (left), and quantification of protein bands (right, *n* = 4).

DHE staining demonstrated that FO therapy could reduce the oxidative response in animals that had received Ang II (Figure 5A). In Ang II-infused animals, the expression of oxidative marker NOX2 and NOX4 (Figure 5B). Similarly, in Ang II-infused mice, FO reduced the production of NOX4 protein which are involved in oxidative signaling cascade (Figure 5C). The TUNEL assay results demonstrated that Ang II infusion increased the incidence of apoptosis compared to that in animals that received saline infusions, and the effect was alleviated by FO (Figure 5D). The expression of Sirt 1, p53, and Bcl-2 was then measured. In contrast to saline-infused mice, Ang II-infused mice exhibited increased expression of p53 and decreased expression of Sirt 1 and Bcl-2, but FO therapy attenuated these responses (Figure 5E). These findings indicated that Sirt1 deubiquitination by USP22 overexpression could prevent p53 transcriptional activation, although this work had significant limitations. Future research will need to confirm the link between USP22 and Sirt 1 and their impact on p53 in basic myocardial cells.

### The decrease in USP22 aggravated Ang II-induced apoptosis, and FO rescued this effect

**FIGURE 6:**
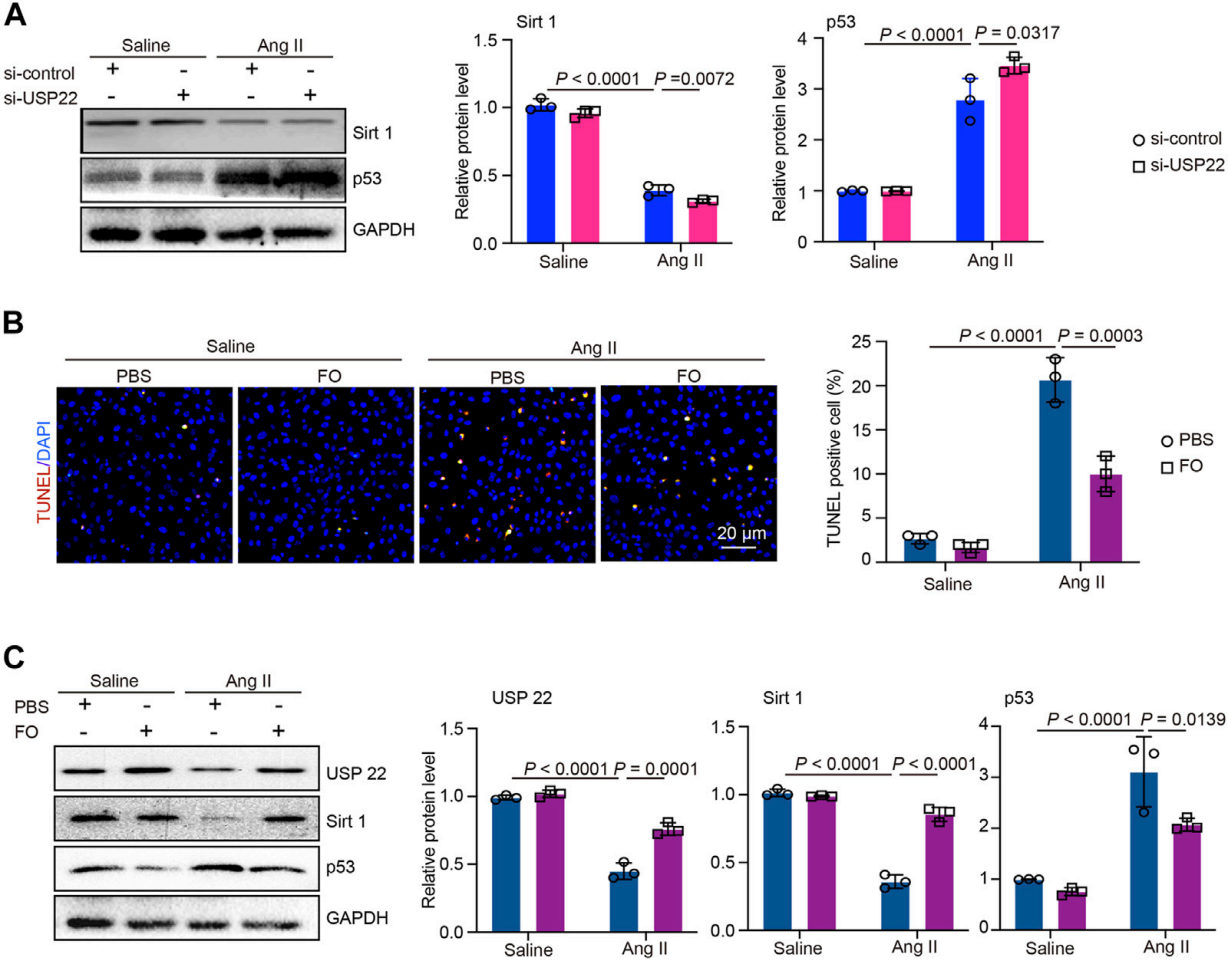
si-USP22 aggravated Ang II-induced apoptosis in AC-16 cells and FO could protect against this effect. AC-16 cells were starved for 2 h and then treated with si-USP22 and si-control for 4h, and then treated with Ang II (100 nM) or saline for 24 h. Similarly, the cells were starved for 2 h and then treated with FO (60 μg/mL) for 4h, and then treated with Ang II (100 nM) or saline for 24 h. **(A)** Western blot analysis of Sirt 1and p53 protein expression in cells (left), and quantification of protein bands (right, *n* = 3); **(B)** TUNEL staining of cells in each group (left, *n* = 3), the quantification of TUNEL positive cell (right, *n* = 3); **(C)** Western blot analysis of USP 22, Sirt 1and p53 protein expression in cells (left), and quantification of protein bands (right, *n* = 3).

To further investigate the role of USP22 and the protective effect of FO, we treated AC-16 cells with si-USP22 and si-control. We found that after treatment with Ang II, the expression of Sirt 1 was significantly decreased, and the addition of si-USP22 aggravated the decrease in Sirt 1 expression induced by Ang II (Figure 6A). In contrast, the expression of p53 was increased by Ang II infusion, and si-USP22 treatment further increased the expression of p53 (Figure 6A). Next, we treated the cells with FO (60 μg/mL). The TUNEL assay showed that Ang II infusion increased apoptosis in AC-16 cells and that FO treatment obviously protected against this reaction (Figure 6B). Furthermore, we detected the expression of USP22, Sirt 1 and p53, and the results showed that Ang II infusion decreased the expression of USP22 and Sirt 1 and increased the expression of p53 compared with the saline group (Figure 6C). FO treatment rescued the decrease in USP22 and Sirt 1 and the increase in p53 induced by Ang II infusion (Figure 6C). These results suggested that the lack of USP22 could induce apoptosis in Ang II-treated cells and that FO treatment could protect against this reaction.

## Discussion

**FIGURE 7:**
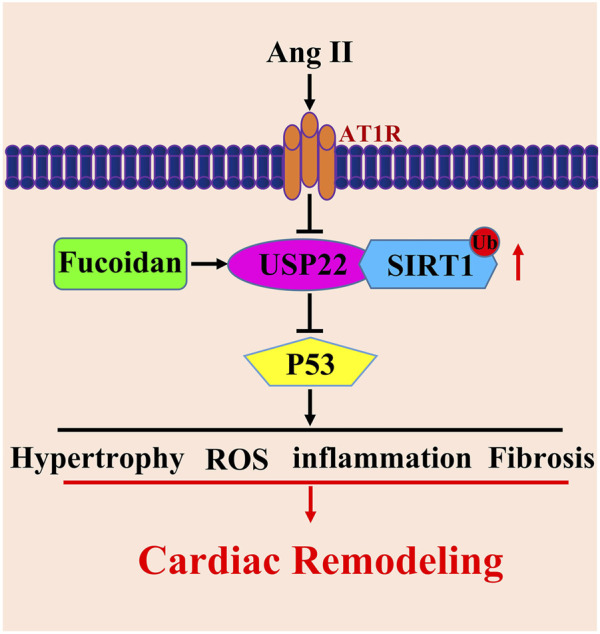
Working model for USP22 in the regulation of Ang II-induced cardiac remodeling. Ang II infusion induced hypertrophy, fibrosis, oxidative stress and inflammasome stimulation which resulted in cardiac remodeling through downregulation of USP22. FO could rescue those reactions. USP22 might by a new sight of heart failure therapy and FO might be used as an agent to protect against Ang II-induced cardiac remodeling.

In the current study, we show that the overexpression of USP22 induced by treatment with FO greatly improved Ang II-induced cardiac dysfunction, heart hypertrophy, inflammation, and fibrosis (Figures 1–4). Additionally, by deubiquitinating Sirt1, the increased expression of USP22 decreased the frequency of p53-dependent apoptosis and oxidative response (Figures 5, 6). Therefore, as indicated in the working model shown in Figure 7, our findings suggest that FO is a targeted approach to treat heart failure, but more clinical trials are required to establish its clinical use and verify its safety.

An increase in cardiac myocyte size without cell division is a characteristic of cardiac hypertrophy. This condition is believed to be an adaptive reaction to increased cardiac afterload-induced wall stress (Zhu et al., 2019). One of the major factors contributing to morbidity and mortality in elderly individuals is cardiac insufficiency, and the etiology of this condition is frequently linked to myocardial remodeling caused by myocardial hypertrophy (Shimizu and Minamino, 2016). The myocardium is impacted by Ang II, which also encourages the development of hypertension. Heart failure and abnormal hypertrophy can result from Ang II exposure. There is evidence that some cell types, such as cardiomyocytes, cardiac fibroblasts, kidney cells, and neurons, are negatively impacted by high intracellular Ang II levels (Zhou et al., 2016). These effects are linked to organ damage, cardiac hypertrophy and fibrosis, conduction problems, and inflammation. They include the promotion of hypertrophy, apoptosis, oxidative stress, and the production of TGF-β and nuclear factor kappa-B (NF-κB) (Benigni et al., 2010). Our earlier research revealed that the ubiquitin‒proteasome system is crucial for controlling protein quality and the prevalence of ventricular fibrillation (Li et al., 2018; Li et al., 2019). Ubiquitin-specific processing proteases (USPs), which are also known as ubiquitin-binding proteins in yeast, are the largest subfamily of deubiquitinases. USP22 is one of these proteins (UBP). From yeast to vertebrates, USP22 is remarkably conserved (Melo-Cardenas et al., 2016). USP22 can stabilize the Sirt 1 protein, which inhibits p53 transcriptional activity and causes cell death, because Sirt1 is polyubiquitinated and targeted for proteasomal degradation. Indeed, abolishing Sirt1 ubiquitination by replacing the ubiquitin-conjugating lysine residues with arginines prolongs Sirt1 half-life. USP22 removes polyubiquitination of Sirt1 to control its protein stability and functions. Therefore, USP22 is a positive regulator of Sirt1. (Lin et al., 2012). Recent research has shown that USP22 protects against myocardial ischemia‒reperfusion injury (Ma et al., 2020), but it is still unknown how USP22 affects Ang II-induced cardiac remodeling and hypertrophy. In the current study, we found that an increase in USP22 could ameliorate Ang II-induced cardiac dysfunction and remodeling.

Marine algae contain large amounts of non-starch polysaccharides that cannot be digested completely by the human digestive system and which therefore have potential as new sources of dietary fiber, prebiotics or other functional ingredients (Kim KT et al., 2014). As with plant fiber from other sources, seaweed fiber is interesting because its consumption has been associated with a significant reduction of chronic diseases such as diabetes, obesity, blood pressure, and so on (Ou et al., 2001; Maki et al., 2007). Soluble fiber can slow down digestion and absorption of nutrients by increasing viscosity and might thereby decrease blood sugar and cholesterol (Kim KT et al., 2014). In this context, FO, a bioactive polysaccharide found in brown algae, appears promising. Marine algae have been considered as a source of enzyme inhibitors. Similar to plant extracts, algal extracts may be considered for this purpose because they contain some polyphenolic compounds, such as bromophenols (Liu et al., 2011) and phlorotannins, which are inhibitors of a-glucosidase. Additionally, polysaccharides isolated from algae have become attractive in the biomedical area because of their numerous bioactivities. Studies have found that FO was an efficient inhibitor of α-amylase and α-glucosidase (Kim KT et al., 2014). The structural changes, molecular weight and concentrations of FO may result in the seasonal variation of a-amylase and α-glucosidase inhibitory activity by FO. Additionally, the structure of FO varies depending on the alga source and could impact the enzyme activity (Kim KT et al., 2014; Pozharitskaya ON et al., 2020). The pharmacokinetics and tissue distribution of this agent are crucial in understanding its biological activity. The microdetermination of fucoidan distribution is one of the key problems in pharmacokinetic studies. Studies have shown that after oral administration, serum levels of FO were increased at 6 h and 9 h (Pozharitskaya ON et al., 2018). The recently described observations of the clinical efficacy of orally administered FO for chronic renal failure indicate probable systemic uptake in humans (Tokita Y et al., 2010). Systemic uptake after oral delivery indicates the potential for additional clinical applications in the future, perhaps including the control of thrombosis. FO’s distinctive biological structure is thought to be the cause of its exceptional biological function. Antioxidant, antitumor, anticoagulant, antithrombotic, immunomodulatory, antiviral, and anti-inflammatory processes are some of the traditional biological processes associated with FO (Luthuli et al., 2019). According to recent research, FO could decrease neutrophil and macrophage accumulation, the level of inflammatory cytokines and lung fibrosis (Yu HH et al., 2018). Furthermore, another study showed that FO could increase the expression and activity of the Sirt 1 protein by upregulating the expression of USP22 (Yu et al., 2020). On the other hand, by downregulating USP22, the RAS system can reduce Sirt 1 protein expression (Wang et al., 2021). In our study, after treatment with FO, the expression of USP22 was markedly increased, and the increase in USP22 increased the activity of Sirt1 and decreased myocardial cell death. FO also reduced macrophage accumulation, oxidative stress and fibrosis in Ang II-induced hypertrophic cardiac remodeling. This approach provides a potential targeted strategy to treat heart failure.

However, our research still has many limitations. We used a single dose of FO treatment, and ubiquitinated Sirt1 expression after USP22 knockdown needs to be examined. We will study more details of FO in our future research.

## Data Availability

The original contributions presented in the study are included in the article/Supplementary Materials, further inquiries can be directed to the corresponding author.
