# Supplementary material for: Fucoidan inhibits apoptosis and improves cardiac remodeling by inhibiting p53 transcriptional activation through USP22/Sirt 1
Source: Front Pharmacol. 2023 May 30;14:1164333. doi: 10.3389/fphar.2023.1164333 (PMC10261984; doi:10.3389/fphar.2023.1164333)
Supplement: Supplementary file 1 [file DataSheet1.DOCX]

**Figure 1A mRNA of USP22**

|  | Saline | 7 days | 14 days |
| --- | --- | --- | --- |
| 1 | 1.011 | 0.763 | 0.612 |
| 2 | 0.987 | 0.742 | 0.534 |
| 3 | 0.945 | 0.755 | 0.677 |
| 4 | 1.021 | 0.813 | 0.578 |
| 5 | 1.112 | 0.911 | 0.701 |
| 6 | 0.879 | 0.849 | 0.679 |

**Figure 1B protein expression of USP22**


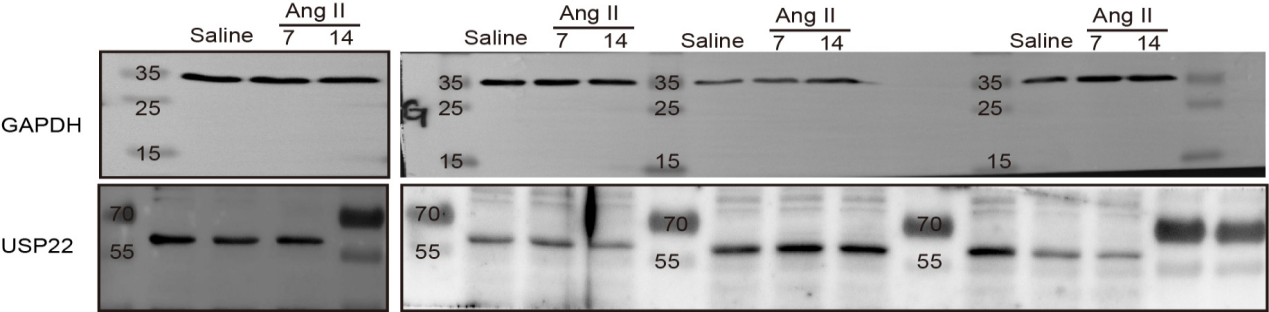


|  | Saline | 7 days | 14 days |
| --- | --- | --- | --- |
| 1 | 1.011 | 0.763 | 0.612 |
| 2 | 0.987 | 0.742 | 0.534 |
| 3 | 0.945 | 0.755 | 0.677 |
| 4 | 1.021 | 0.813 | 0.578 |
| 5 | 1.112 | 0.911 | 0.701 |
| 6 | 0.879 | 0.849 | 0.679 |

**Figure 2B SBP**

|  | Saline+PBS | | | | | | Saline+FO | | | | | |
| --- | --- | --- | --- | --- | --- | --- | --- | --- | --- | --- | --- | --- |
| -1 | 101 | 95 | 94 | 103 | 86 | 105 | 101 | 104 | 105 | 102 | 94 | 105 |
| 0 | 96 | 87 | 83 | 84 | 107 | 106 | 89 | 93 | 103 | 93 | 96 | 92 |
| 2 | 83 | 103 | 94 | 84 | 103 | 104 | 98 | 105 | 105 | 102 | 99 | 104 |
| 4 | 92 | 92 | 91 | 107 | 95 | 90 | 89 | 106 | 94 | 104 | 105 | 102 |
| 6 | 103 | 109 | 92 | 91 | 83 | 83 | 97 | 95 | 93 | 104 | 99 | 93 |
| 8 | 98 | 100 | 105 | 90 | 97 | 105 | 102 | 105 | 97 | 109 | 91 | 100 |
| 10 | 82 | 93 | 90 | 88 | 88 | 82 | 88 | 93 | 89 | 89 | 85 | 98 |
| 12 | 101 | 84 | 110 | 87 | 98 | 108 | 102 | 89 | 84 | 98 | 102 | 101 |
| 14 | 98 | 99 | 98 | 102 | 101 | 95 | 83 | 96 | 96 | 99 | 94 | 112 |

|  | Ang II+PBS | | | | | | Ang II+FO | | | | | |
| --- | --- | --- | --- | --- | --- | --- | --- | --- | --- | --- | --- | --- |
| -1 | 105 | 92 | 93 | 93 | 97 | 96 | 104 | 95 | 96 | 101 | 102 | 101 |
| 0 | 97 | 106 | 112 | 89 | 106 | 95 | 98 | 102 | 103 | 110 | 103 | 102 |
| 2 | 119 | 107 | 99 | 125 | 117 | 118 | 106 | 96 | 122 | 101 | 104 | 114 |
| 4 | 129 | 132 | 156 | 122 | 132 | 133 | 111 | 103 | 98 | 106 | 109 | 110 |
| 6 | 140 | 145 | 133 | 140 | 132 | 132 | 116 | 116 | 107 | 111 | 104 | 105 |
| 8 | 152 | 134 | 134 | 153 | 136 | 151 | 119 | 106 | 115 | 122 | 116 | 124 |
| 10 | 141 | 147 | 137 | 148 | 151 | 145 | 106 | 106 | 113 | 118 | 122 | 125 |
| 12 | 139 | 141 | 165 | 166 | 161 | 155 | 119 | 106 | 115 | 122 | 116 | 124 |
| 14 | 164 | 159 | 144 | 143 | 152 | 154 | 105 | 115 | 107 | 123 | 129 | 127 |

**Figure 2C**


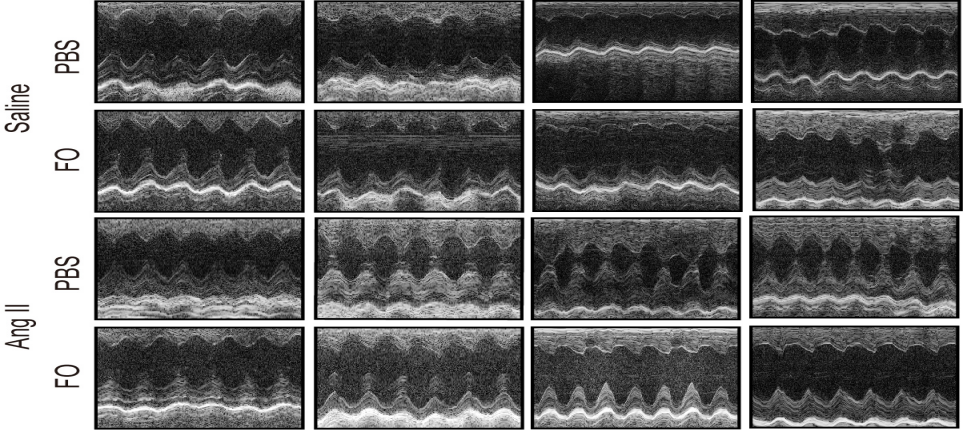


**Figure 2D**

EF%

|  | PBS | | | | FO | | | |
| --- | --- | --- | --- | --- | --- | --- | --- | --- |
| Saline | 58.38 | 61.97 | 67.36 | 55.49 | 63.29 | 63.78 | 57 | 54.46 |
| Ang II | 77.19 | 76.1 | 79.55 | 74.73 | 68.11 | 54.29 | 67.68 | 59.92 |

FS%

| PBS | | | | | | | FO | | |
| --- | --- | --- | --- | --- | --- | --- | --- | --- | --- |
| Saline | 32.61 | 36.42 | 28.18 | 26.16 | 33.6 | 34.16 | | 29.26 | 27.91 |
| Ang II | 46.15 | 42.33 | 41.33 | 46.81 | 37.16 | 27.37 | | 42.11 | 30.61 |

**Figure 2E**


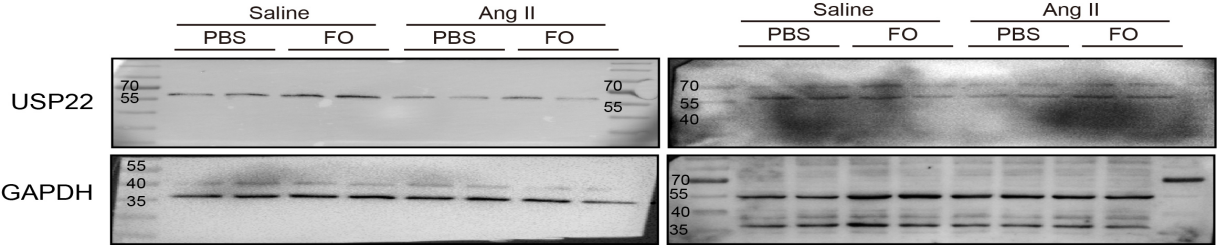


| USP22 | PBS | | | | FO | | | |
| --- | --- | --- | --- | --- | --- | --- | --- | --- |
| Saline | 1.012 | 0.998 | 1.007 | 1 | 1.211 | 1.098 | 1.213 | 1.154 |
| Ang II | 0.766 | 0.745 | 0.632 | 0.597 | 0.896 | 0.874 | 0.91 | 0.845 |

**Figure 3A**

H & E


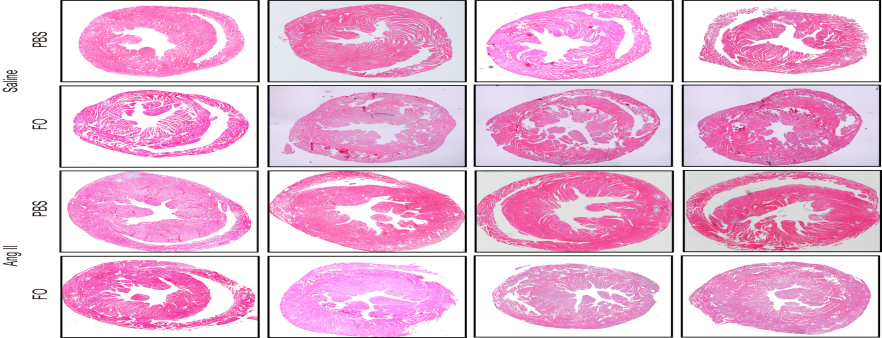


| HW/BW | | | | | | | | | PBS | | | | | | | | | | FO | | | | | | | | | |  |
| --- | --- | --- | --- | --- | --- | --- | --- | --- | --- | --- | --- | --- | --- | --- | --- | --- | --- | --- | --- | --- | --- | --- | --- | --- | --- | --- | --- | --- | --- |
| Saline | 4.53 | | 5.06 | | 4.51 | | 4.68 | | | | 4.32 | | 4.71 | | 4.49 | | 4.62 | | | | 4.72 | | 4.64 | | 4.52 | | 4.43 | |  |
| Ang II | 6.55 | | 6.43 | | 5.78 | | 5.43 | | | | 5.82 | | 6.29 | | 4.78 | | 4.68 | | | | 5.01 | | 4.98 | | 5.11 | | 5.08 | |  |
| HW/TL | | | | | | | | | | PBS | | | | | | | | | | FO | | | | | | | | | |
| Saline | | 7.49 | | 6.8 | | 6.93 | | 6.42 | | | | 5.52 | | 6.89 | | 6.92 | | 7.03 | | | | 6.82 | | 6.75 | | 6.35 | | 6.8 | |
| Ang II | | 8.25 | | 8.62 | | 8.5 | | 7.98 | | | | 8.12 | | 8.18 | | 7.32 | | 7.54 | | | | 7.96 | | 7.37 | | 7.73 | | 7.4 | |

**Figure 3C**


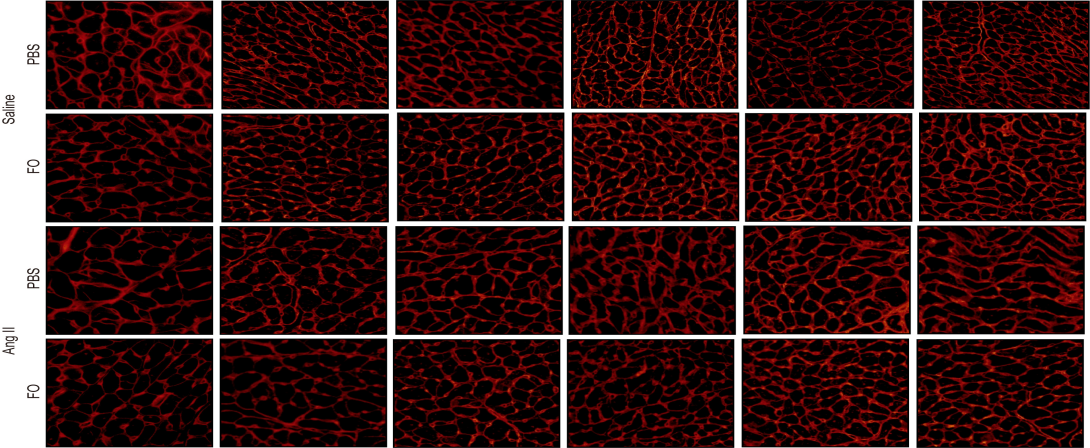


|  | PBS | | | | | | FO | | | | | |
| --- | --- | --- | --- | --- | --- | --- | --- | --- | --- | --- | --- | --- |
| Saline | 0.725 | 1.012 | 1.179 | 1.174 | 1.082 | 0.829 | 0.783 | 0.874 | 1.011 | 0.864 | 0.913 | 0.766 |
| Ang II | 2.840697002 | 2.3997027 | 2.519448344 | 3.231150384 | 2.463952432 | 2.34916178 | 1.795854323 | 1.538359898 | 1.497398629 | 1.701379139 | 1.341811875 | 1.509125444 |

**Figure 3D DHE**


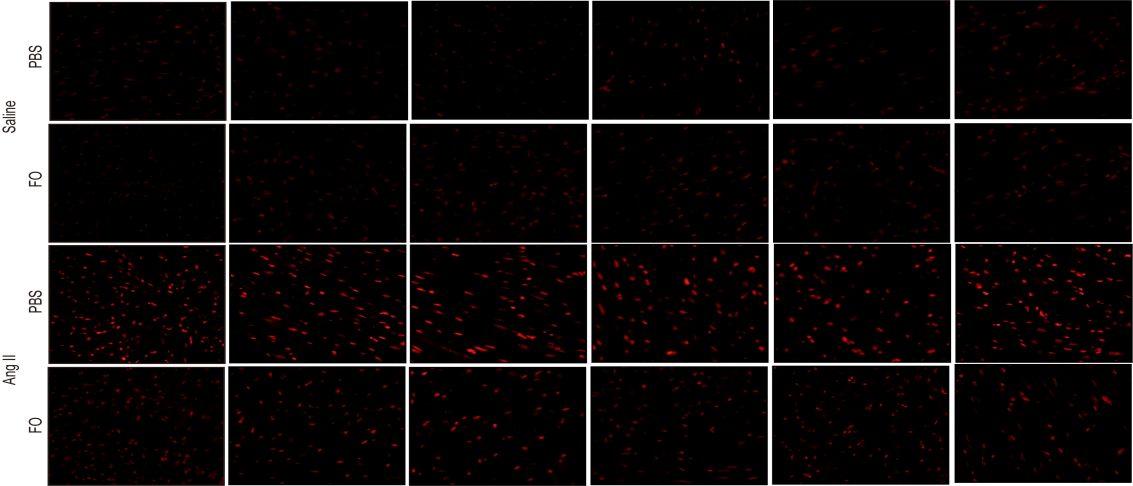


| DHE | PBS | | | | | | FO | | | | | |
| --- | --- | --- | --- | --- | --- | --- | --- | --- | --- | --- | --- | --- |
| Saline | 1.07 | 1.045 | 0.978 | 0.876 | 0.912 | 1.134 | 1.011 | 0.877 | 0.932 | 0.847 | 0.913 | 0.945 |
| Ang II | 3.233 | 2.785 | 2.012 | 2.413 | 1.987 | 1.966 | 1.542 | 1.457 | 1.329 | 1.201 | 1.731 | 1.649 |

**Figure 3E PCR ANF BNP**

| ANF | PBS | | | | | FO | | | |
| --- | --- | --- | --- | --- | --- | --- | --- | --- | --- |
| Saline | 0.878 | 0.904 | 1.09 | 1.019 | | 0.871 | 1.211 | 0.751 | 1.172 |
| Ang II | 4.207 | 2.006 | 2.87 | 2.915 | | 0.669 | 1.758 | 2.559 | 1.511 |
| BNP | PBS | | | | FO | | | |  |
| Saline | 0.955 | 0.752 | 0.727 | 0.992 | 0.747 | 0.744 | 0.702 | 1.095 |  |
| Ang II | 3.343 | 2.661 | 2.952 | 4.025 | 1.405 | 1.082 | 1.259 | 1.588 |  |

**Figure 3F PCR NOX2 NOX4**

| NOX2 | PBS | | | | | FO | | | |
| --- | --- | --- | --- | --- | --- | --- | --- | --- | --- |
| Saline | 0.765 | 1.296 | | 0.918 | 1.152 | 0.656 | 0.718 | 1.152 | 0.809 |
| Ang II | 2.2951 | 3.426 | | 2.7764 | 2.6957 | 1.416 | 1.046 | 1.149 | 1.301 |
| NOX4 | PBS | | | | FO | | | |  |
| Saline | 0.881 | 0.863 | 1.105 | 1.07 | 0.852 | 0.844 | 1.192 | 0.881 |  |
| Ang II | 2.318 | 2.658 | 2.66 | 2.403 | 1.2478 | 0.7005 | 1.5338 | 1.1508 |  |

**Figure 3G**


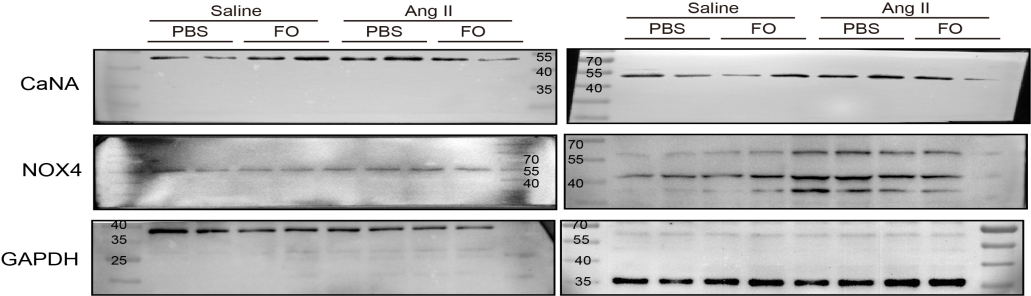


| CaNA | PBS | | | | FO | | | |
| --- | --- | --- | --- | --- | --- | --- | --- | --- |
| Saline | 0.985 | 1.106 | 0.974 | 1.012 | 1.213 | 1.011 | 0.896 | 0.932 |
| Ang II | 1.798 | 2.013 | 1.974 | 2.102 | 1.841 | 1.322 | 1.601 | 1.023 |

| NOX4 | PBS | | | | FO | | | |
| --- | --- | --- | --- | --- | --- | --- | --- | --- |
| Saline | 0.896 | 0.911 | 0.942 | 1.011 | 0.876 | 1.132 | 0.876 | 0.978 |
| AngII | 1.344 | 1.401 | 1.376 | 1.432 | 1.301 | 1.005 | 0.986 | 0.874 |

**Figure 4A**

H&E


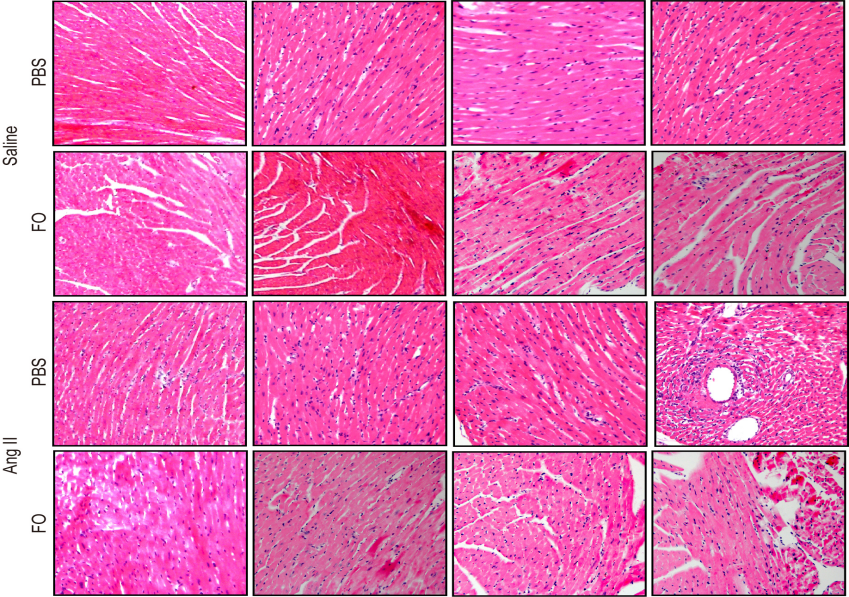


CD68


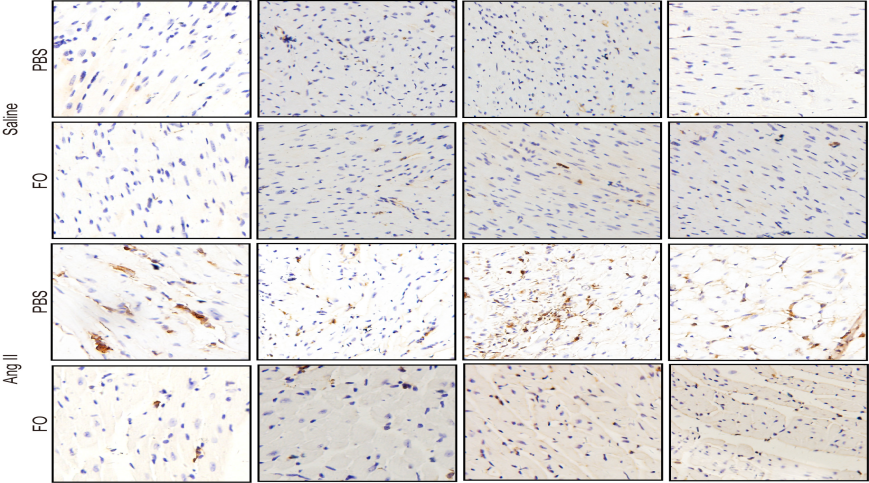


| CD68 | PBS | | | | | | FO | | | | | |
| --- | --- | --- | --- | --- | --- | --- | --- | --- | --- | --- | --- | --- |
| Saline | 3 | 2 | 4 | 3 | 1 | 5 | 2 | 3 | 3 | 2 | 1 | 2 |
| Ang II | 10 | 9 | 9 | 8 | 12 | 9 | 3 | 5 | 4 | 3 | 5 | 4 |

**Figure 4B**

Masson


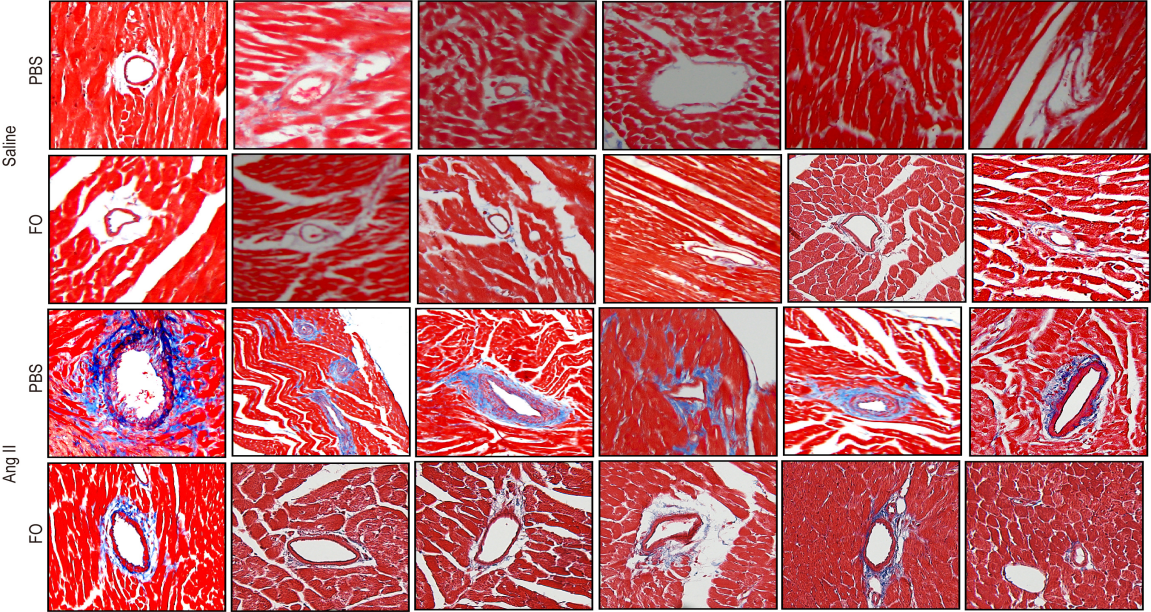


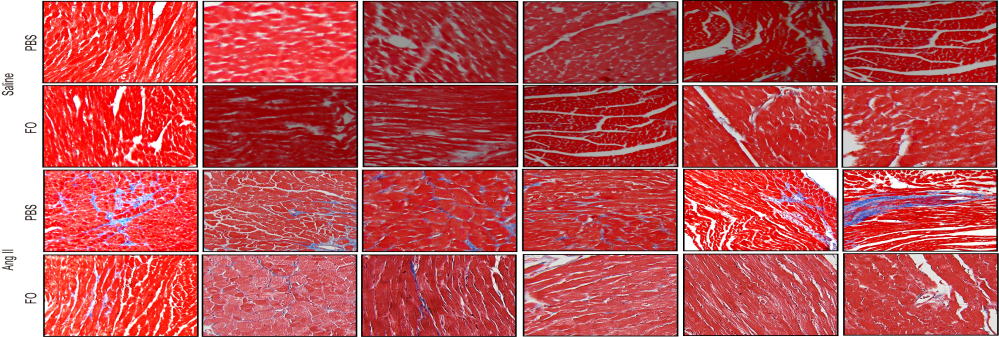


| Masson | PBS | | | | | | FO | | | | | |
| --- | --- | --- | --- | --- | --- | --- | --- | --- | --- | --- | --- | --- |
| Saline | 1.021 | 0.986 | 0.887 | 1.103 | 1.002 | 0.969 | 0.832 | 0.944 | 0.937 | 0.845 | 1.032 | 1.015 |
| Ang II | 4.312 | 3.987 | 4.574 | 4.189 | 3.969 | 4.01 | 1.577 | 2.015 | 1.996 | 1.874 | 1.945 | 1.739 |

**Figure 4C PCR**

| IL-1β | PBS | | | | | | | | FO | | | | | | | | |
| --- | --- | --- | --- | --- | --- | --- | --- | --- | --- | --- | --- | --- | --- | --- | --- | --- | --- |
| Saline | 0.90265 | | 1.07294 | | 1.12451 | | 0.89374 | | 0.863326664 | | 0.776986758 | | 0.648096646 | | 0.99627437 | | |
| Ang II | 2.089110878 | | 3.016397304 | | 2.260811979 | | 1.876386223 | | 1.59927223 | | 1.172090244 | | 1.051520101 | | 0.72462101 | | |
| IL-6 | | PBS | | | | | | | | FO | | | | | | |  |
| Saline | | 1.049608442 | | 1.187008297 | | 0.850104494 | | 0.913368967 | | 0.811891 | | 0.786031 | | 0.527672 | | 1.190053 |  |
| Ang II | | 1.67761 | | 4.74231 | | 3.90577 | | 3.30419 | | 1.375 | | 0.801 | | 1.347 | | 1.081 |  |

**Figure 4D PCR**

Collagen I

|  | PBS | | | | FO | | | |
| --- | --- | --- | --- | --- | --- | --- | --- | --- |
| Saline | 0.999706165 | 1.356796238 | 0.661854176 | 0.981886041 | 1.1509295 | 0.926299381 | 0.710725655 | 0.964717999 |
| Ang II | 1.546036929 | 1.998915438 | 1.554750325 | 1.935411029 | 0.955926909 | 1.152554885 | 1.172883753 | 0.854244938 |

Collagen III

|  | PBS | | | | FO | | | |
| --- | --- | --- | --- | --- | --- | --- | --- | --- |
| Saline | 0.86188599 | 1.231394453 | 0.736221628 | 1.172125982 | 1.069586491 | 0.851617796 | 1.018291846 | 0.852025751 |
| Ang II | 2.495046596 | 1.774126762 | 2.220852022 | 2.333315426 | 1.231951955 | 0.841859024 | 1.672138337 | 1.327492822 |

**Figure 4E WB**


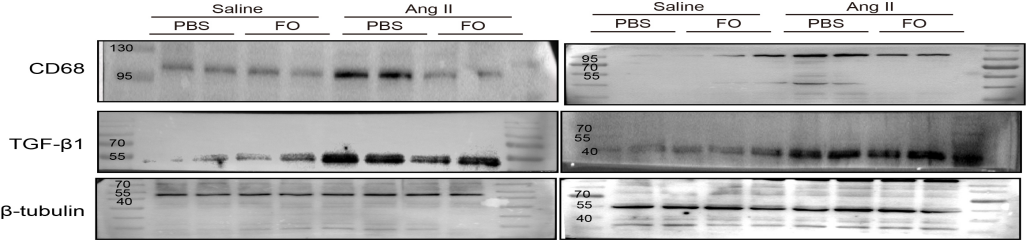


| CD68 | PBS | | | | FO | | | |
| --- | --- | --- | --- | --- | --- | --- | --- | --- |
| Saline | 1.012 | 0.986 | 0.811 | 0.932 | 0.935 | 1.011 | 0.825 | 0.921 |
| Ang II | 2.345 | 2.476 | 3.011 | 2.978 | 0.834 | 1.211 | 0.945 | 1.3401 |

| TGF-β | PBS | | | | FO | | | |
| --- | --- | --- | --- | --- | --- | --- | --- | --- |
| Saline | 1.001 | 0.978 | 0.932 | 1.1 | 1.101 | 0.823 | 1.045 | 0.989 |
| Ang II | 3.422 | 4.035 | 4.152 | 4.243 | 3.561 | 2.987 | 2.745 | 2.821 |

**Figure 5A TUNEL**


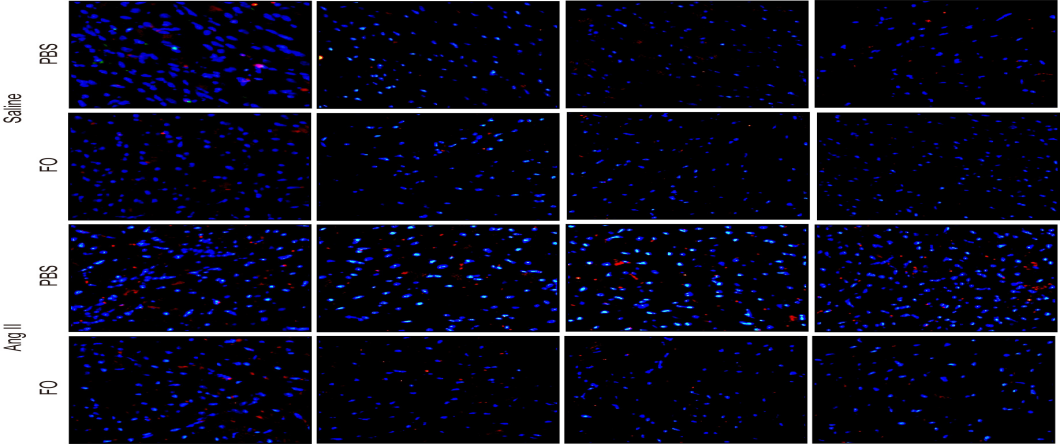


| TUNEL | PBS | | | | | | FO | | | | | |
| --- | --- | --- | --- | --- | --- | --- | --- | --- | --- | --- | --- | --- |
| Saline | 3 | 4 | 4 | 5 | 3 | 3 | 2 | 3 | 2 | 3 | 3 | 4 |
| Ang II | 15 | 16 | 21 | 15 | 17 | 20 | 8 | 9 | 10 | 12 | 8 | 9 |

**Figure 5B WB**


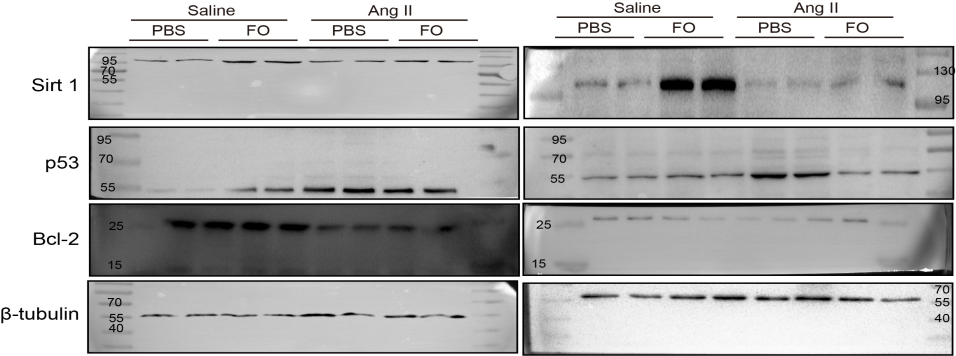


| Sirt 1 | PBS | | | | FO | | | |
| --- | --- | --- | --- | --- | --- | --- | --- | --- |
| Saline | 1.013 | 0.998 | 1.045 | 0.876 | 1.541 | 1.321 | 1.297 | 1.404 |
| Ang II | 0.876 | 0.745 | 0.732 | 0.698 | 1.062 | 0.978 | 0.996 | 1.101 |

| p53 | PBS | | | | FO | | | |
| --- | --- | --- | --- | --- | --- | --- | --- | --- |
| Saline | 1.001 | 0.978 | 0.899 | 1.012 | 1.301 | 1.201 | 1.1 | 1.211 |
| Ang II | 3.333 | 3.405 | 2.911 | 3.612 | 1.574 | 1.677 | 1.813 | 1.574 |

| Bcl2 | PBS | | | | FO | | | |
| --- | --- | --- | --- | --- | --- | --- | --- | --- |
| Saline | 1.001 | 1.012 | 0.987 | 0.932 | 0.931 | 1.012 | 0.988 | 0.976 |
| Ang II | 0.512 | 0.534 | 0.607 | 0.432 | 0.761 | 0.732 | 0.641 | 0.733 |

**Figure 6A**


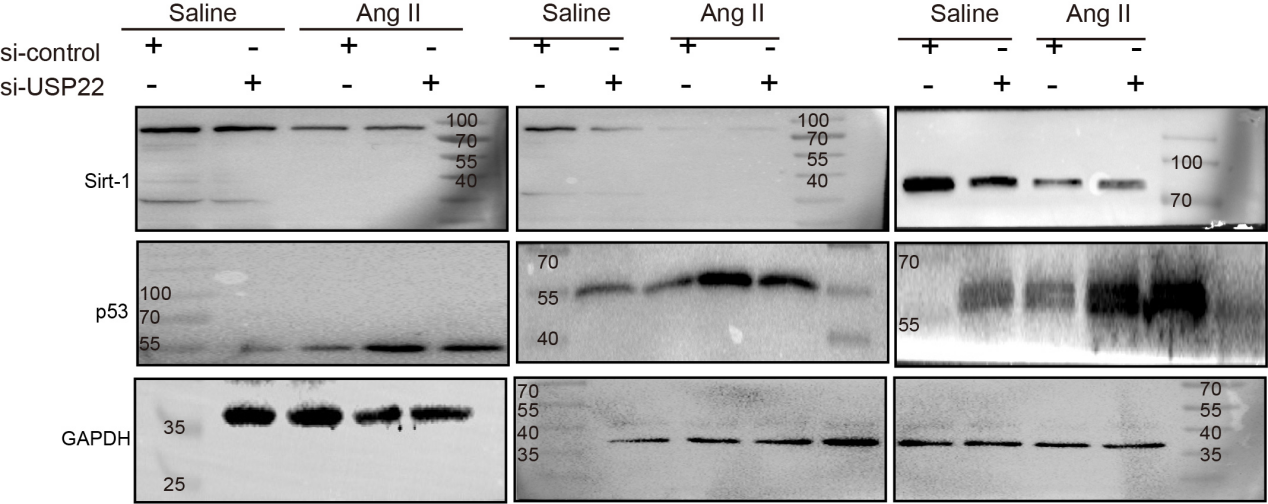


| Sirt 1 | si-control | | | si-usp22 | | |
| --- | --- | --- | --- | --- | --- | --- |
| Saline | 1.071 | 0.987 | 1.005 | 0.976 | 0.923 | 0.978 |
| Ang II | 0.347 | 0.402 | 0.423 | 0.315 | 0.323 | 0.298 |

| p53 | si-control | | | si-usp22 | | |
| --- | --- | --- | --- | --- | --- | --- |
| Saline | 1.01 | 0.998 | 0.979 | 0.988 | 1 | 0.996 |
| Ang II | 2.375 | 3.212 | 2.784 | 3.412 | 3.336 | 3.645 |

**Figure 6B**


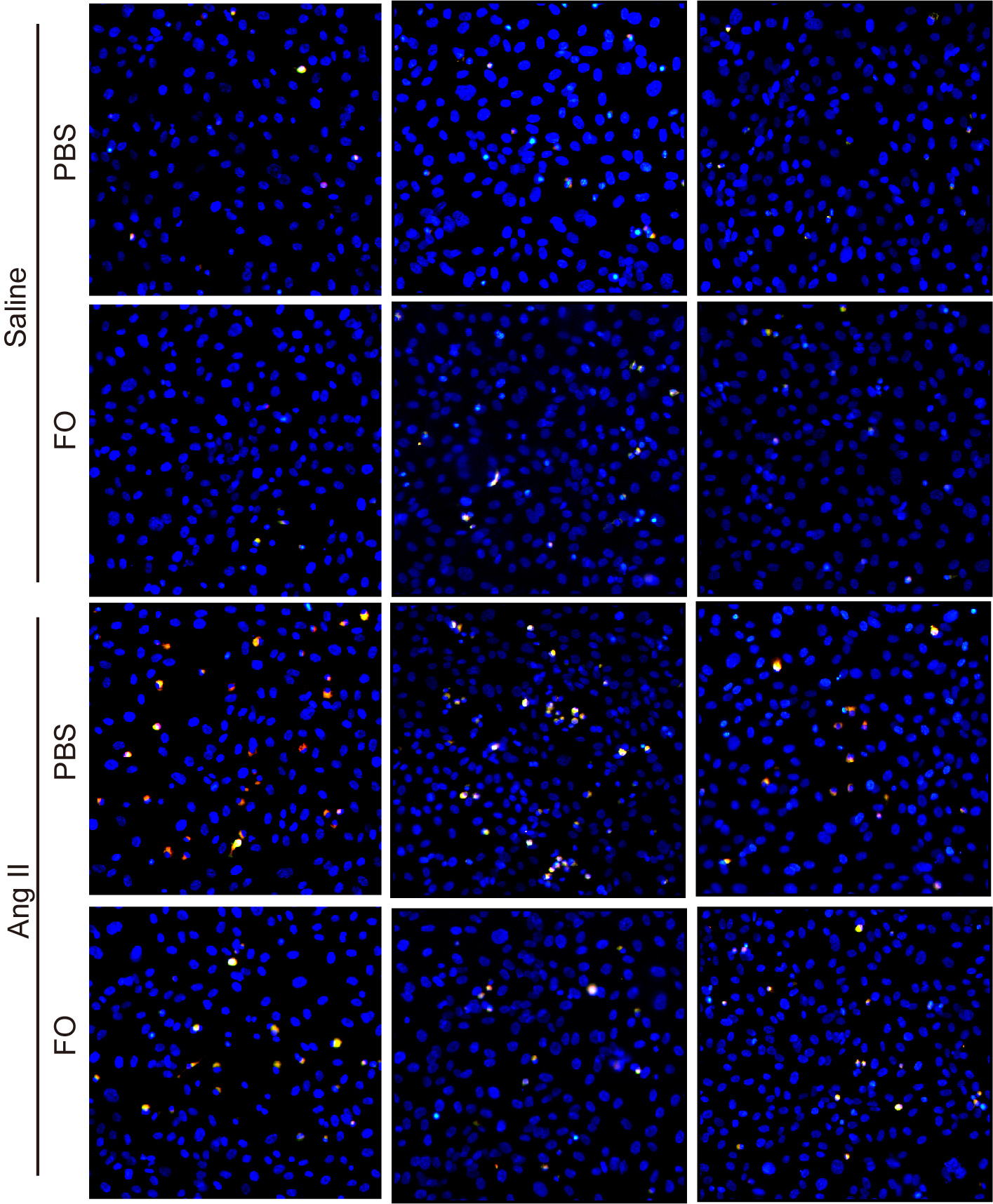


| TUNEL | PBS | | | FO | | |
| --- | --- | --- | --- | --- | --- | --- |
| Saline | 3 | 2 | 3 | 2 | 1 | 2 |
| Ang II | 21 | 18 | 23 | 10 | 8 | 12 |

**Figure 6C**


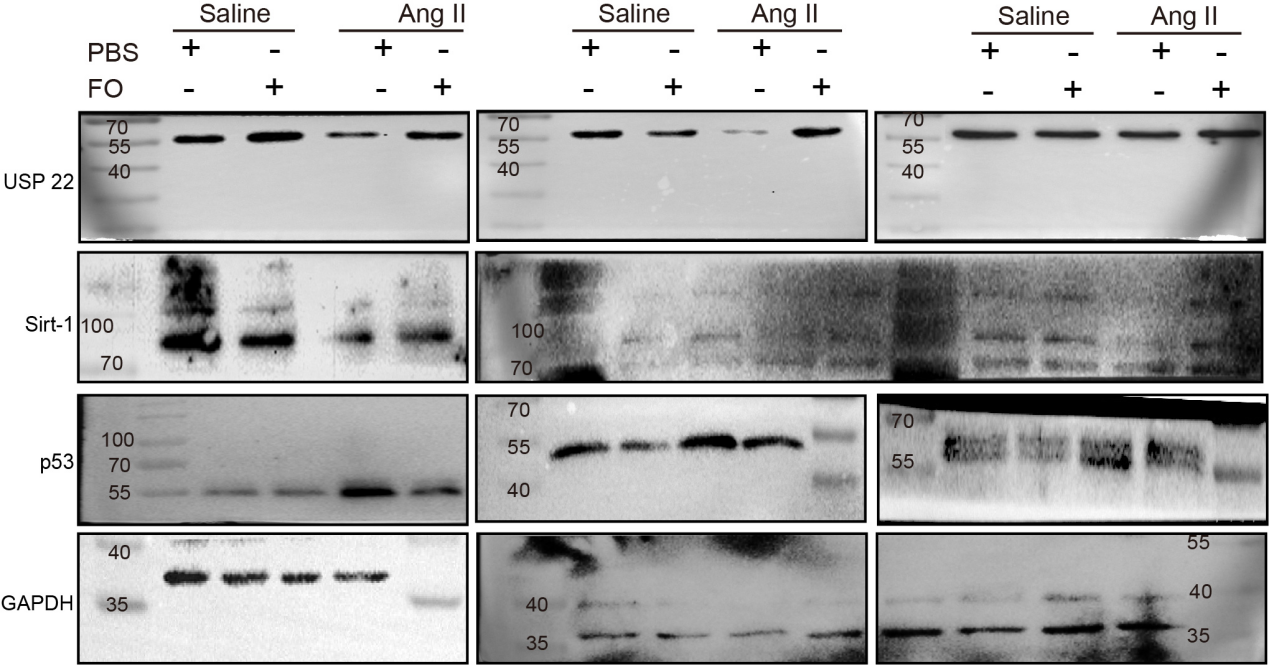


| Usp22 | PBS | | | FO | | |
| --- | --- | --- | --- | --- | --- | --- |
| Saline | 1.011 | 0.976 | 0.989 | 0.993 | 1.021 | 1.045 |
| Ang II | 0.401 | 0.432 | 0.518 | 0.723 | 0.811 | 0.745 |

| Sirt 1 | PBS | | | FO | | |
| --- | --- | --- | --- | --- | --- | --- |
| Saline | 1.045 | 1 | 0.997 | 0.987 | 0.996 | 0.985 |
| Ang II | 0.312 | 0.411 | 0.356 | 0.874 | 0.895 | 0.798 |

| P53 | PBS | | | FO | | |
| --- | --- | --- | --- | --- | --- | --- |
| Saline | 1 | 0.996 | 0.989 | 0.754 | 0.832 | 0.679 |
| Ang II | 2.312 | 3.467 | 3.545 | 2.011 | 1.987 | 2.214 |
